# Mutations to Medicine: Advances in Medical Management of Paediatric, Adolescent, and Young Adult Low-Grade Gliomas

**DOI:** 10.3390/cancers18203363

**Published:** 2026-10-17

**Authors:** Ian Burns, Julie Bennett

**Affiliations:** 1 Division of Haematology/Oncology, Hospital for Sick Children, Toronto, ON M5G 1X8, Canada; julie.bennett@sickkids.ca; 2 Department of Paediatrics, University of Toronto, Toronto, ON M5G 1X8, Canada; 3 Arthur and Sonia Labatt Brain Tumor Research Centre, Hospital for Sick Children, Toronto, ON M5G 1X8, Canada; 4 Division of Medical Oncology and Hematology, Princess Margaret Cancer Centre, Toronto, ON M5G 2C4, Canada

**Keywords:** low-grade glioma, paediatric cancer, AYA cancer, CNS tumours, targeted therapy, BRAF-altered LGG, IDH-mutant LGG

## Abstract

Gliomas are the most common brain tumours in children, adolescents, and young adults. In children, paediatric-type low-grade gliomas (pLGG) predominate, while IDH-mutant low-grade gliomas (LGG) become more common in young adulthood. While outcomes with complete surgical resection are excellent, many pLGG patients with residual or unresectable tumours experience significant long-term functional impairment despite conventional medical treatment. IDH-mutant LGG have an unfavourable natural history, with high risk of malignant transformation. With the emergence of precision medicine, targeted therapies have offered hope as more effective treatments. For pLGG, favourable short-term outcomes have been demonstrated with MAPK inhibitors. Significant challenges remain, however, including designing treatment regimens that prevent regrowth, understanding mechanisms and biomarkers of response and resistance, and addressing treatment-related toxicities. Furthermore, pLGG manifest as a chronic disease, and it remains unclear whether targeted therapies can yield superior long-term functional outcomes relative to conventional chemotherapy. For IDH-mutant LGG, IDH inhibitors hold promise; however, it is unknown whether monotherapy will avert malignant transformation. Notably, adolescent and young adult patients have been excluded from many clinical trials, resulting in limited data on the efficacy and toxicity of pLGG-directed targeted therapies in this population. Clinical trials and patient care for LGG should be designed around tumour biology rather than patient age. Overall, the molecular era has transformed our understanding of LGG and facilitated the development of novel therapies. The next challenge is to translate new biological insights into patient- and tumour-specific treatment regimens that can improve long-term disease control and functional outcomes.

## Simple Summary

With the identification of the driver alterations of low-grade gliomas in children, adolescents, and young adults, novel targeted therapies have emerged and transformed the treatment landscape for these tumours. Initial data for these therapies are promising; however, more work is needed to understand whether they can yield more durable tumour control and improve long-term functional outcomes relative to conventional therapies. In this review, we summarize key recent advances in the medical management of low-grade gliomas in children, adolescents, and young adults, highlight persistent challenges, and identify potential strategies to improve long-term outcomes.

## 1. Introduction

Gliomas are the most common brain tumours in children, adolescents, and young adults (AYA, ages 15–39) [1,2]. In paediatrics, paediatric-type low-grade gliomas (pLGG) predominate [2,3,4]. In AYA, approximately one quarter are pLGG, while half are isocitrate dehydrogenase (IDH)-mutant astrocytomas or oligodendrogliomas [5].

pLGG are clinically heterogeneous, occurring across the central nervous system (CNS) with a range of histologies, growth trajectories [3,5], and functional outcomes [6,7]. In the last two decades, molecular characterization of pLGG has revealed that the vast majority are driven by aberrant rat sarcoma virus (RAS)/mitogen-activated protein kinase (MAPK) signalling, with the most common driver alterations being *KIAA1549::BRAF*, BRAF p.V600E, and *NF1* alterations, which together account for approximately 2/3 of pLGG. The next most common drivers are fibroblast growth factor receptor *(FGFR)* alterations (most commonly *FGFR1::TACC1*, tyrosine kinase duplications [TKD], and single nucleotide variants [SNV]), followed by other rare alterations resulting in RAS/MAPK upregulation [3]. The distribution of pLGG drivers changes with age, with a higher proportion of fusion-driven pLGG in paediatrics compared with SNV-driven pLGG in AYA [5]. Malignant transformation of pLGG is rare overall but more common in those driven by BRAF p.V600E [8].

The long-term survival for pLGG patients is excellent, with approximately 90% alive beyond 20 years from diagnosis [3,9,10]. pLGG therefore have the potential to manifest as a chronic disease. While many patients can be cured with surgical resection alone [11] and experience few sequelae, there remains significant morbidity in patients with more troublesome lesions, such as those in deep-seated regions and/or eloquent regions like the optic pathway [6,7,12,13,14,15,16]. Importantly, disseminated pLGG are unique clinical entities with poorer long-term survival and can have inferior responses to therapy [3,17,18].

IDH-mutant gliomas are distinct from pLGG. These tumours rarely occur in children—in a large multi-institutional analysis, they comprised only 0.5% of gliomas in patients <10 years old [4]. The incidence of these tumours steadily increases with age and peaks in the AYA range [5]. They can be low-grade (WHO grade 2) or high-grade (WHO grade 3 or 4), and are driven by a mutation in *IDH*, with IDH-1 p.R132H being most common, followed by other hotspot mutations in *IDH-1* and *IDH-2* [5]. Long-term survival is much poorer for patients with IDH-mutant gliomas relative to pLGG, as they typically transform into high-grade lesions over time [5,19].

In this review, we discuss how precision medicine has transformed the medical management of LGG, from treatment in the pre-molecular era to the emergence of targeted therapies. We review the evidence around key targeted therapies, the importance of functional outcomes in assessing treatment efficacy, potential biomarkers, and the unique challenges faced when caring for AYA with LGG. Finally, we summarize persistent challenges, unanswered questions, and potential strategies to achieve durable tumour control and improved functional outcomes for children and AYA with LGG. Relevant literature was identified through secondary sources, PubMed searches, and based on existing knowledge of the field. Findings from conference abstracts were included if additive to peer-reviewed research.

## 2. Medical Management in the Pre-Molecular Era

Maximal safe resection is the foundation of low-grade glioma management. When gross total resection is achieved, pLGG have a very low risk of progression. For example, 8-year progression-free survival (PFS) of 93% was reported for a large cohort of such patients [11]. Extent of resection is also associated with superior overall survival and PFS in IDH-mutant gliomas [20]. Unfortunately, gross total resection is not feasible for many patients, and incompletely resected tumours, which are frequently located in midline or eloquent areas, often require adjuvant therapy. Prior to the molecular era, the two available adjuvant therapies were radiotherapy and chemotherapy.

While efficacious, radiotherapy is associated with significantly increased long-term mortality [9,10] and other late effects such as cognitive deficits, vasculopathy, and endocrinopathies [6,21,22]. Therefore, over the last several decades, chemotherapy has become the standard-of-care adjuvant therapy for pLGG, both at diagnosis and recurrence.

The most commonly used chemotherapy regimens for pLGG include the combination of vincristine and carboplatin, vinblastine monotherapy, and the combination of thioguanine, procarbazine, lomustine, and vincristine (TPCV). Prospective trials overall have shown similar survival outcomes, with 5-year PFS of approximately 30–50% across regimens [23,24,25,26,27]. Neurofibromatosis type 1 (NF1)-associated pLGG have also been demonstrated to have superior PFS relative to sporadic pLGG [26,27]. Most prospective studies of chemotherapy for pLGG are limited by a lack of long-term follow-up and do not include stratification by driver alteration.

As first-line chemotherapy is unable to achieve long-term disease control in more than half of pLGG patients, many receive treatment with another line of chemotherapy at relapse. Some have reported similar rates of progression with chemotherapy at relapse compared with first-line chemotherapy [28,29], while others have reported poorer outcomes [30]. Inferior PFS for non-NF1 patients treated with vinblastine at relapse relative to platinum-based chemotherapy has also been observed [30].

Chemotherapy for pLGG is generally well-tolerated, with bone marrow suppression being the most common toxicity across regimens. Other notable toxicities include carboplatin hypersensitivity reactions and vincristine-associated peripheral neuropathy [25]. Vinblastine monotherapy is considered to be better tolerated than vincristine/carboplatin and TPCV [31].

For IDH-mutant low-grade gliomas, extent of surgical resection also plays a significant role in prognosis [20]. Treatment can be deferred depending on this and other prognostic factors, including histology, molecular features, and age, given the toxicity of therapy and inevitability of malignant transformation. For those in need of therapy, chemotherapy and/or radiotherapy have been used either upfront or at progression [19].

Overall, while well-tolerated, chemotherapy does not achieve long-term disease control for a substantial portion of pLGG, and many patients experience long-term disease-related morbidity, emphasizing the ongoing need for alternative approaches to yield more durable control and improve functional outcomes. Given the propensity of IDH-mutant LGG to transform to high-grade lesions, there is also an urgent need to identify therapies that can alter this dire natural history. The molecular characterization of these tumours has facilitated the development of novel therapies that have the potential to achieve these goals.

## 3. Overview of Targeted Therapies for LGG

**Figure 1:**
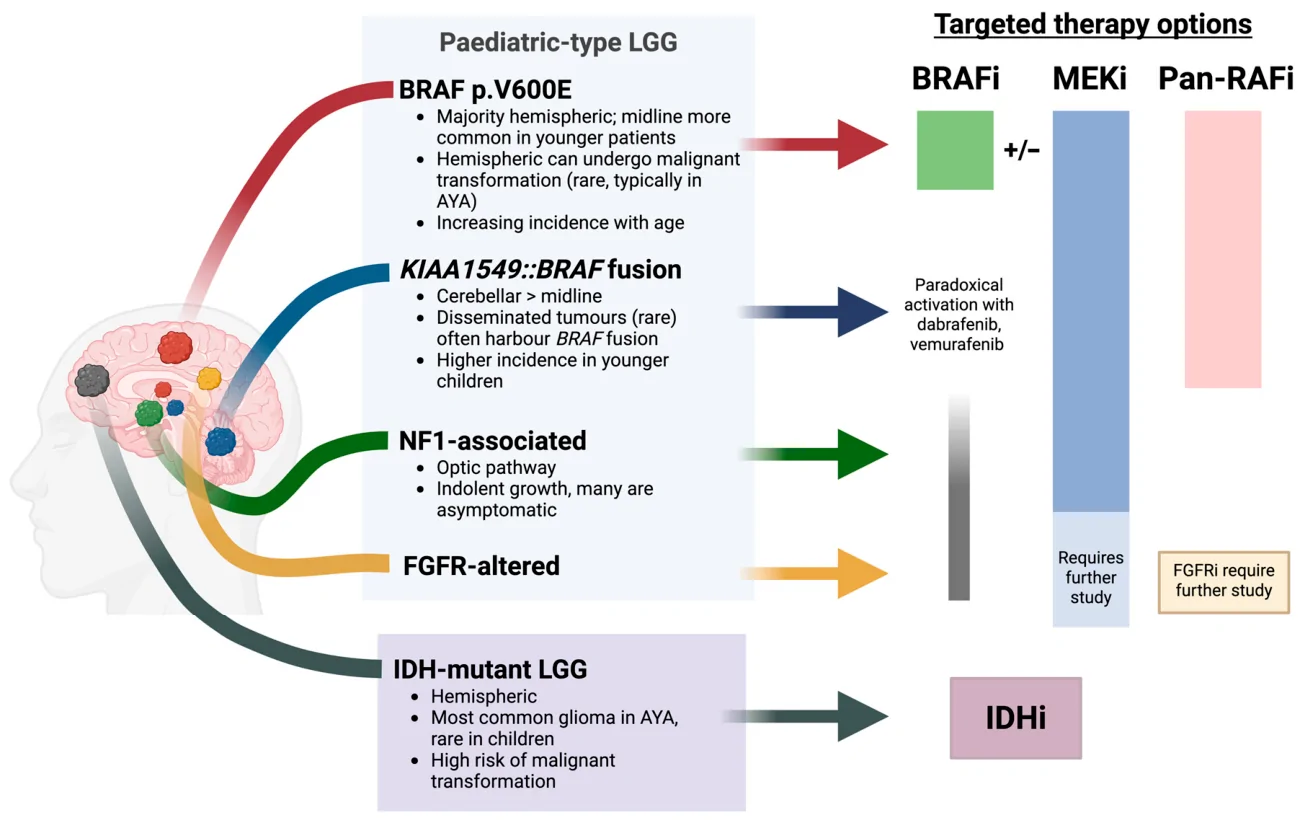
Targeted therapy options for paediatric and AYA low-grade gliomas. Abbreviations: LGG, low-grade glioma; AYA, adolescent and young adult; BRAFi, BRAF inhibitor; MEKi, MEK inhibitor; Pan-RAFi, Pan-RAF inhibitor; IDHi, IDH inhibitor; FGFRi, FGFR inhibitor. References: [3,5,17,32,33,34]. Created in BioRender. Burns, I. (2026) https://BioRender.com/ii2yp2g (accessed on 8 October 2026).

Studies of targeted therapies for LGG to date have demonstrated promising radiologic responses and short-term outcomes, but it remains unclear how best to implement targeted therapies to yield durable long-term tumour control, minimize associated toxicities, and optimize functional outcomes (Figure 1, Table 1).

### 3.1. BRAF Inhibitors

Approximately 20% of sporadic pLGG are driven by BRAF p.V600E [3], and *BRAF* mutations are also enriched in AYA pLGG relative to other driver alterations [5]. In recent years, BRAF inhibitors, including dabrafenib and vemurafenib, have begun to replace chemotherapy in the upfront management of BRAF-mutant pLGG. In early-phase trials, partial responses (≥50% reduction in size) were seen in 30–45% of participants [35,45]. In a retrospective cohort of 56 pLGG treated with BRAF inhibition, 80% of tumours achieved a minor response (≥25% reduction) or better. Notably, there is a risk of rapid progression upon drug discontinuation [46].

Importantly, first-generation BRAF inhibitors (including dabrafenib and vemurafenib) should be avoided in tumours that lack a BRAF p.V600 mutation as they can paradoxically activate the MAPK pathway by inducing RAF dimerization [47,48]. Newer generation drugs are under study to overcome this phenomenon, as discussed below.

Given evidence from the adult solid tumour literature for greater efficacy and fewer adverse events with dabrafenib combined with the MEK1–2 inhibitor trametinib, this combination has been studied and is now FDA-approved for use as upfront therapy in BRAF p.V600E pLGG for patients <18 years of age [36,49]. This approval is based on a phase 2 randomized trial comparing upfront combination dabrafenib/trametinib with vincristine/carboplatin. It included 110 patients aged 1–17 years and demonstrated superior tumour responses and a PFS benefit in those treated with targeted therapy, with a median follow-up of 18.9 months. Targeted therapy was well-tolerated, with lower rates of grade 3 toxicities and treatment discontinuation than observed with chemotherapy [36].

Unfortunately, there has been limited study of this drug combination in patients over 18 years old with BRAF-mutated LGG. The ROAR basket trial evaluated a small cohort of 13 such patients and demonstrated promising responses and short-term PFS [38,50]. It is uncertain, however, whether there are differences in tumour responses, disease control, and toxicities between AYA and paediatric patients treated with dabrafenib with or without trametinib.

There is ongoing discussion regarding the use of BRAF inhibitor monotherapy versus combination with trametinib as initial targeted therapy. Given that excellent responses can be seen with BRAF inhibition alone, the Canadian paediatric neuro-oncology community has suggested that it may be appropriate to start monotherapy in some patients. This strategy could improve cost-effectiveness, improve compliance, and, in the case of progression, a MEK inhibitor could be added [51,52].

### 3.2. MEK Inhibitors

MEK is downstream of BRAF in the RAS/MAPK pathway, making it a promising target for *BRAF*-fused, *NF1*-driven, and RAS/MAPK-driven pLGG. MEK inhibitors include selumetinib, trametinib, mirdametinib, binimetinib, cobimetinib, and pimasertib.

Selumetinib has been well-studied across BRAF-altered and NF1-associated pLGG, with partial response rates up to 40% (≥50% reduction) and 2-year PFS of approximately 70% and 96%, respectively [39,40]. Importantly, not all patients respond to therapy, and in fact, approximately one-third of patients with BRAF-altered tumours progressed on therapy [39,40]. Thirty-five patients were retreated with selumetinib after therapy discontinuation, all of whom had shown stability or response with the initial course. With retreatment, a lower response rate of 14% was reported in preliminary data; however, an encouraging 5-year PFS of 56% was observed [53].

Several trials of selumetinib are ongoing, including comparing it to vincristine/carboplatin as upfront therapy (NCT04166409) and alone or in combination with vinblastine for recurrent/progressive pLGG (NCT04576117).

Trametinib monotherapy has also been evaluated and shown efficacy, though published data are mostly limited to retrospective cohorts [29,37,54,55,56,57]. The TRAM-01 trial, a phase 2 study of trametinib for recurrent/progressive pLGG with activation of the RAS/MAPK pathway, enrolled 68 patients (majority *BRAF*-fused). A preliminary objective response rate (≥25% reduction) of 46% and promising short-term PFS have been reported, though many patients required another therapeutic intervention after planned discontinuation of the drug after 72 weeks of treatment [58]. As with selumetinib, studies have shown that a subset of tumours are resistant or refractory to treatment [29,54,58]. Trametinib is currently being explored as upfront therapy in comparison with vinblastine (NCT05180825).

Other MEK inhibitors currently under active study include binimetinib (NCT06159478) [59] and mirdametinib [60]. The MIRV trial will evaluate mirdametinib as upfront therapy in combination with vinblastine (NCT06666348).

While MEK inhibition has been most extensively explored in tumours driven by *BRAF* fusions, NF1 alterations, and BRAF p.V600E, there are also reports of efficacy for FGFR-altered pLGG [58,61]. A 2025 review identified eight FGFR-altered pLGG treated with MEK inhibition, reporting one partial response, six tumours with stable disease, and one progression [61].

### 3.3. Next-Generation MAPK Inhibitors

Several next-generation MAPK inhibitors are also under study and may have advantages over earlier generations, including efficacy for tumours refractory/resistant to other inhibitors, a lack of paradoxical activation, and different toxicity profiles.

Tovorafenib is a pan-RAF inhibitor that has activity against BRAF alterations that generate oncogenic signalling from monomers (including BRAF p.V600E) and via dimerization (*BRAF* fusions). Efficacy has been demonstrated in a phase 2 study of relapsed BRAF-altered pLGG, with an objective response rate (≥25% reduction) of 51%. Response was independent of type of BRAF alteration, and notably 33% of patients who had progressed during prior MAPK inhibitor therapy showed an objective response to tovorafenib [42]. Interestingly, a recent abstract reported that 77% of patients remained treatment-free for ≥12 months after therapy discontinuation, and investigators observed minimal rebound growth [62]. These preliminary single-arm data suggest that responses may be more durable after discontinuation of tovorafenib relative to BRAF and MEK inhibitors; it will be important to perform appropriate comparative studies to elucidate differences between MAPK inhibitors. Based on these results, tovorafenib has been FDA-approved for use in BRAF-aberrant relapsed/refractory pLGG and is under evaluation as monotherapy in the upfront setting in comparison with standard-of-care chemotherapy (NCT05566795), and for relapsed/refractory pLGG in combination with vinblastine (NCT06381570) [63].

Other next-generation agents under evaluation include plixorafenib, a BRAF inhibitor designed to avoid paradoxical RAS/MAPK activation through disruption of BRAF dimerization (NCT05503797), and avutometinib, a dual RAF and MEK inhibitor (NCT06104488).

### 3.4. ERK Inhibitors

ERK is downstream of MEK in the RAS/MAPK pathway, and in addition to potential use as monotherapy, ERK inhibition may have a synergistic effect when combined with other MAPK inhibitors [64]. To date, there have been limited studies of ERK inhibition [65]. Ulixertinib, an ERK1/2 inhibitor, is currently under evaluation in a window-of-opportunity trial for RAS/MAPK-activated gliomas in patients >12 years of age (NCT05804227).

### 3.5. MAPK Inhibitor-Associated Toxicities

MAPK inhibitors have overall been well-tolerated in prospective trials to date. Several toxicities, however, have emerged as key clinical concerns, and the long-term toxicity profiles of these agents remain unclear. Additionally, given that treatment durations can be prolonged or indefinite, even mild toxicities may have a significant effect on tolerability and quality of life.

Prominent in the patient experience are cutaneous toxicities, including paronychia, dermatitis, acneiform reactions, and other rashes [32]. While the rates of specific cutaneous toxicities vary between MAPK inhibitors, nearly all treated patients will experience some degree of toxicity [66]. For example, with selumetinib, various CTCAE grade 1–2 dermatologic toxicities were reported in more than half of patients [39]. In their retrospective cohort, Green et al. reported that 80% of MAPK inhibitor-treated patients had at least one grade ≥2 dermatologic toxicity [54]. In the phase 2 randomized trial of dabrafenib and trametinib versus chemotherapy, dermatologic toxicity was higher with targeted therapy, though few patients had resultant dose reduction or interruption [36]. Multidisciplinary and guideline-based care for the proactive management of these toxicities is essential to maximize tolerability of therapy [66].

Other common toxicities include fatigue, pyrexia, weight gain, endocrinopathies, hyponatremia, edema, gastrointestinal symptoms, liver enzyme elevation, creatine kinase elevation, and bone marrow suppression. Notable rare serious toxicities include cardiotoxicity with reduced ejection fraction and ocular concerns including uveitis and retinal detachment [32,67]. Tovorafenib has some notable distinct toxicities including reduced growth velocity (with many patients, especially at younger ages, recovering after discontinuation) and hair colour change. Serious intratumoural hemorrhage was noted in five (4% of total) patients in the phase 2 study of tovorafenib, with three discontinuing treatment. The relative contributions of tovorafenib versus the natural history of some tumours to hemorrhage remain unclear. Less pyrexia, diarrhea, and weight gain were seen with tovorafenib relative to other MAPK inhibitors, along with no ocular or cardiotoxicity [42,68].

### 3.6. FGFR Inhibitors

To date, the data for use of FGFR inhibition in FGFR-altered pLGG are sparse, and these agents require further evaluation. A recent meta-analysis of FGFR-altered pLGG treated with FGFR inhibition (majority erdafitinib) included 24 patients: four demonstrated partial responses, 15 stable disease, and five progressive disease [61]. Importantly, responses to FGFR inhibition have been demonstrated in patients with both *FGFR* fusions and mutations [33]. In contrast to other pLGG, there is a high rate of co-alterations in downstream MAPK components in *FGFR1* SNV-driven pLGG, which may contribute to the development of resistance to monotherapy [33]. It may be that unique combination therapies are required to achieve durable responses in FGFR-altered tumours. Additionally, FGFR inhibitors have unique toxicities, including significant ophthalmologic toxicities (often dose limiting), hyperphosphatemia, dermatologic concerns, and diarrhea. There is also a specific risk of bone toxicity in skeletally immature patients [33].

### 3.7. mTOR Inhibitors

Limited efficacy relative to other targeted therapies has been demonstrated in trials of mammalian target of rapamycin (mTOR) inhibitor monotherapy for relapsed pLGG, with stable disease being the best response for most patients and 30–50% of patients progressing within 1 year of starting therapy [69,70,71]. Everolimus is currently under investigation in combination with trametinib for recurrent gliomas (NCT04485559).

### 3.8. IDH Inhibitors

IDH inhibition holds promise as a novel strategy for the medical management of IDH-mutant LGG. In a landmark phase 3 randomized, double-blind, placebo-controlled trial of vorasidenib for residual or recurrent non-enhancing grade 2 IDH-mutant glioma, patients treated with vorasidenib demonstrated significantly longer PFS, time to next intervention, and reduced tumour growth velocity. Notably, no patients under 18 years old were treated with vorasidenib in this trial. Vorasidenib was overall well-tolerated. Approximately one quarter of patients experienced a grade 3 or higher adverse event, most commonly alanine aminotransferase elevation, and 12% experienced a serious adverse event, most commonly seizures (which were also seen in patients on the placebo arm) [43,44]. Other IDH inhibitors are also under study for both low-grade and high-grade IDH-mutant gliomas, including olutasidenib (NCT06161974) [72] and safusidenib (NCT04458272). Work is also ongoing to evaluate IDH inhibition in combination with other agents such as immune checkpoint inhibitors (NCT04056910, NCT05484622).

These exciting findings have raised several important questions. First, there are scenarios where it is unclear if there is a role for IDH inhibitors. This includes low-grade tumours in patients with gross total resections, patients with low-grade tumours that have previously been treated with chemoradiation without indication of malignant transformation, and those with higher-grade lesions. Additionally, it is uncertain whether IDH inhibition can alter the natural history of eventual malignant transformation seen in these gliomas. Finally, the long-term toxicities from these therapies are unknown [34,73].

## 4. Optimizing Long-Term Functional Outcomes

pLGG are a chronic disease—most patients survive, so the goal of medical management is to improve long-term functional outcomes, optimize quality of life during and after treatment, and minimize long-term toxicity of therapy. By virtue of their design and follow-up times, prospective trials of treatment for pLGG lack long-term data to measure the trajectory of the disease over the span of a lifetime. The tumour response data and short-term PFS reported in these trials provide limited insight into the long-term impact of the tumour and associated therapies. Despite these limitations, retrospective data indicate that long-term functional outcomes for patients treated with conventional therapies have been poor. It is uncertain whether targeted therapies can improve this outlook; consequently, investigation of their impact on long-term functional outcomes should be prioritized in ongoing and future studies.

A key functional outcome is vision, as optic pathway gliomas make up a large proportion of tumours that require medical treatment [25,27]. In a cohort of 81 children with optic pathway glioma under the age of 3 at diagnosis, long-term visual outcomes were poor, despite most patients undergoing treatment with chemotherapy [13]. Similarly, in another retrospective study with a median of 5.2 years of follow-up, more than half of patients with sporadic optic pathway gliomas had severe vision loss in at least one eye [16].

It is currently unclear how treatment with targeted therapy can impact vision in the long term, especially as currently available data on the impact of targeted therapy on vision are predominantly in the relapsed setting (by which time there is a higher chance of irreversible decline) and limited by short follow-up. Short-term data from a randomized trial showed that 34% of eyes in patients treated with upfront dabrafenib/trametinib showed improved visual acuity, versus only 11% in the chemotherapy group [36]. With selumetinib treatment, visual improvement was noted in 21% of recurrent/progressive non-NF1 optic glioma patients, with a further 68% showing stability with treatment [41]. Tovorafenib was also shown to yield similar rates of stability and improvement in relapsed/refractory BRAF-altered optic pathway glioma patients [74]. In a retrospective cohort of 35 optic pathway gliomas treated with MAPK-targeted therapy at relapse, there was visual benefit in 72% of patients; however, most (63%) achieved stable vision rather than improvement [54]. It has been suggested that early intervention with targeted therapies or bevacizumab could be applied as a sight-saving strategy in at-risk patients, but this requires prospective evaluation [13].

An added challenge in the assessment of visual responses in the paediatric population is that many patients present at an age that limits objective evaluation of key outcomes such as visual acuity and visual fields. The current recommendation by the RAPNO working group is to follow the REiNS recommendations for visual assessment with Teller acuity cards or HOTV, with a 0.2 logMAR change as the cutoff for improvement/worsening [75,76].

Despite poorer long-term survival compared with pLGG, patients with IDH-mutant gliomas can nevertheless survive for many years to decades after diagnosis [5,77]. When considering functional outcomes in the context of these typically hemispheric tumours, it is important to balance minimizing treatment- and tumour-related morbidity with the goal of prolonging survival by staving off malignant transformation.

LGG can also be associated with impairment in motor skills, adaptive behaviour, communication, and other neurocognitive domains [12,78,79]. Trials are now routinely including measures of these areas, along with quality-of-life measures, as secondary or exploratory outcomes. For patients and families, however, functional outcomes have primacy. The evaluation of therapeutic regimens must be grounded in improving short- and long-term function along with quality of life. It is crucial for these studies to include long-term assessment of these key metrics given the anticipated decades of survival.

## 5. Identifying Biomarkers to Enable Personalized Therapy

While the identification of driver alterations was a major step forward in understanding the biology of LGG, there is significant clinical heterogeneity within each alteration group [3,5,17], and this is reflected in heterogeneous responses to treatment. Work to better understand the relationships between tumour biology, tumour microenvironment, and clinical trajectory will allow for more tailored approaches to treatment. Specifically, identification of biomarkers to predict tumour growth potential, response or resistance to therapy, and risk of rebound growth would be the epitome of precision medicine.

Studies of the tumour immune microenvironment have demonstrated heterogeneity between and within tumours of each pLGG driver alteration and that immune cell infiltration can carry prognostic value [80,81,82]. Emerging data also suggest a complex role for microglia in tumour responses to RAS/MAPK inhibition and rebound growth after treatment discontinuation [83,84]. From a therapeutic perspective, anti-programmed cell death protein 1 (PD-1) therapy, which can enhance T-cell anti-tumour activity, has been shown to be effective in combination with MAPK inhibition in melanoma [85], and is now being explored in BRAF- and NF1-altered gliomas (NCT06712875).

Measurement of RAS/MAPK pathway gene activation to predict sensitivity to MAPK inhibition has also shown promise [83]. Additionally, oncogene-induced senescence contributes to the variable growth trajectories of pLGG, and further characterization of factors that affect these pathways may allow for more effective prognostication and novel therapeutic targets such as BCL-XL [86,87].

It will also be important to address the development of resistance to MAPK inhibition, which can occur via a variety of mechanisms including activation of alternative signalling pathways and autophagy [88]. Hydroxychloroquine, which inhibits autophagy, has been investigated in combination with MEK +/− BRAF inhibition in patients with treatment-resistant BRAF-altered glioma, with preliminary data showing that some LGG had improved responses when compared with their prior course of therapy (NCT04201457) [89].

For IDH-mutant LGG, there are many parallels to pLGG, with ongoing efforts to better understand how host and tumour factors influence tumour natural history, especially the trajectory of malignant transformation and response to targeted therapies [34]. For example, it has been proposed that positron emission tomography (PET) imaging could be used to predict susceptibility to IDH inhibition [90,91].

In addition to imaging, the identification of other predictive biomarkers that can be measured non-invasively, such as via liquid biopsy of serum or cerebrospinal fluid, would be impactful. In addition to circulating tumour DNA, which is particularly challenging to detect from LGG, proteomic and metabolomic liquid biopsy analyses may reveal useful biomarkers [92].

## 6. AYA-Specific Challenges in Advancing Treatment for LGG

AYA represent an orphan population in LGG management, with biology that straddles paediatric and adult disease. They are frequently excluded from trials for pLGG, and there are very limited data for targeted therapies in AYA.

For example, the 2023 randomized trial of dabrafenib and trametinib versus chemotherapy only included patients up to 17 years old [36]. The oldest patient in the phase 2 selumetinib trial was 21 years old [39,40,41], and the phase 2 trial of tovorafenib had only nine patients between 15 and 25 years old [42]. Some trials now have broader age cutoffs; however, data on outcomes for AYA pLGG will continue to lag significantly behind paediatric data [34,93]. Due to lack of evidence, many patients are offered treatment based on paediatric evidence that may not be biologically or age-appropriate. Despite sharing driver alterations, pLGG in AYA can have different natural histories than those in children [5] and may show distinct responses to therapy.

Tumour-agnostic trials, such as the ROAR trial, present another opportunity for AYA to be treated on a trial and generate important data [38,50,94]. Unfortunately, there are systematic barriers precluding enrollment in trials, including patient geography, access to molecular testing, access to age- and biology-relevant clinical trials within health care systems, and financial constraints.

The limited data on targeted therapy in AYA also means toxicity data for this group are sparse, and the adult literature suggests that AYA may manifest different side effects with targeted therapy. For example, adults appear to have a particular risk of developing squamous cell carcinomas and keratoacanthomas secondary to MAPK inhibition. Additionally, the higher prevalence of pre-existing comorbidities such as cardiac disease in adults can contribute to different toxicities [95]. Aside from toxicity, the unique psychosocial impacts of LGG in AYA—who are confronting transitions into independent living, parenthood, and careers among other challenges—are also understudied [93].

For those seeking to conceive, targeted therapies should not be used as they may have teratogenic effects. Unfortunately, there are limited data to inform patient management in this scenario. It is uncertain whether the frequency and degree of rebound pLGG growth seen in children [46,96] is similar in AYA, and it is therefore challenging to assess risk with stopping therapy in these patients.

It is crucial that AYA LGG be treated in the context of a clinical trial whenever possible to enable evidence-based advancement in this orphan population. It is also important to ensure access to multi-disciplinary care for AYA based on tumour biology rather than age alone. For example, a virtual pan-Canadian AYA CNS tumour board rounds has been shown to yield a dramatic increase in the number of AYA patients with pLGG who were recommended to start targeted therapy, with only a minority being eligible for a clinical trial [97].

## 7. Future Directions

The short-term prospective data for targeted therapies for LGG are promising, and they are increasingly being used as upfront therapies and at relapse/progression. The combination of dabrafenib and trametinib is FDA-approved for the upfront treatment of BRAF p.V600E pLGG [49], the pan-RAF inhibitor tovorafenib is FDA-approved for relapsed/refractory BRAF-altered pLGG [63], there is strong phase II evidence for MEK inhibition in MAPK-altered pLGG [39,40,41,58], and the IDH inhibitor vorasidenib has been FDA-approved for upfront management of IDH-mutant LGG [98].

Despite these advances, it remains unclear how targeted therapies can be optimally incorporated into treatment regimens to improve long-term outcomes for LGG. What is clear is that for a substantial portion of pLGG patients (particularly those with sporadic pLGG), chemotherapy does not achieve disease control in the long term [31], and morbidity remains high. Against this background, emerging evidence suggests that targeted therapy may yield superior disease control for non-NF1 pLGG compared with chemotherapy [29,36,54]. While NF1-associated pLGG have superior PFS with chemotherapy [26,27], many patients nevertheless experience visual morbidity and may benefit from alternative therapies [13,15,18,99].

Chemotherapy treatment, however, is time-limited and generally well-tolerated [25,27]. Given the risks of rebound growth and progression after discontinuation, treatment with targeted therapy is increasingly prolonged or indefinite. With this time horizon, the burden of treatment rises. Even minor toxicities can affect quality of life, and it remains unknown what toxicities might be associated with long-term treatment. Additionally, treatment costs with prolonged courses can become prohibitive, and therapies may be inaccessible in low- and middle-income countries [52].

While responses are robust with targeted therapy, it is not yet evident whether more significant reductions in tumour size relative to chemotherapy contribute meaningfully to improved long-term functional outcomes. Timing of therapy may also be significant—will early initiation of targeted therapy in patients reduce long-term morbidity such as visual decline? Additionally, there is ongoing debate regarding what defines clinically meaningful disease progression while on or off therapy. For example, if a tumour shrinks significantly with treatment, a relatively small increase in size from the nadir meets the RAPNO definition of radiologic progression of >25% [75] and may not be clinically meaningful. Conversely, for large tumours, progression of <25% may have a profound impact on clinical symptoms and neurologic function.

From a management perspective, we cannot yet reliably predict which tumours within each alteration group will respond to targeted therapy. Additionally, it is not clear how to optimally stop or wean targeted therapies to maximize the chances of maintaining durable disease control. Responses are generally rapid with these therapies, and once responses are achieved, a lower dose of drug may be sufficient to maintain them [100]. Retreatment after discontinuation may also yield less significant responses relative to initial therapy [53,100]. Weaning regimens for patients with BRAF p.V600E pLGG treated with BRAF +/– MEK inhibition are currently under study [51] (NCT07110246). Long-term treatment and weaning must be systematically evaluated to understand how to optimally stop targeted therapy. Identification of relevant biomarkers should be tied to these studies and may allow for regimens to be individually tailored. Additionally, comparative studies between targeted therapies of the same and different classes may identify the most effective agent for specific contexts.

As mentioned previously, combinatorial therapies may yield deeper and more durable responses. In addition to combination with other targeted agents, combinations of MAPK inhibition and chemotherapy are currently being prospectively evaluated (NCT06381570, NCT04576117, NCT06666348).

Novel agents and non-MAPK targets for pLGG should also continue to be explored. For example, a recent study has shown that the BRAF fusion partner KIAA1549 itself is essential for oncogenesis and has unique therapeutic vulnerabilities, highlighting the importance of looking beyond the proto-oncogene when developing targeted therapies for fusion-driven tumours [101].

As with targeted therapies for pLGG, IDH inhibition has transformed the landscape of treatment for IDH-mutant gliomas; however, there remains significant uncertainty around the effects of these agents on tumour natural history, how best to apply them to the clinic, and their long-term toxicities. Next steps include addressing these uncertainties, evaluating treatment regimens that include combining IDH inhibition with conventional therapies and other targeted/immunotherapies, and developing other novel agents [73,102].

## 8. Conclusions

Overall, advancing the management of LGG will require concerted biological and clinical progress. From a biological perspective, greater insight into the heterogeneous natural histories of LGG is needed. This includes characterizing the mechanisms of dissemination, senescence, and transformation, and will require broadening our view beyond tumour biology to include host factors. Better understanding the interplay between tumour/host biology and therapeutic responses will also allow for personalized approaches to treatment. From a clinical research perspective, study eligibility criteria should focus on tumour biology rather than patient age. Comprehensive characterization of functional outcomes over the long term is essential to understand the true impact of therapy, along with ongoing work to define clinically meaningful responses and progression. In clinical practice, clinical trial enrollment should be encouraged whenever feasible. AYA patients should have access to molecularly informed care from those with expertise in managing the specific tumour biology. Ensuring equitable access to care must also remain a priority—efforts to improve drug access and develop cost-effective treatment strategies are essential to ensuring global access to these advances in care.

**Figure 2:**
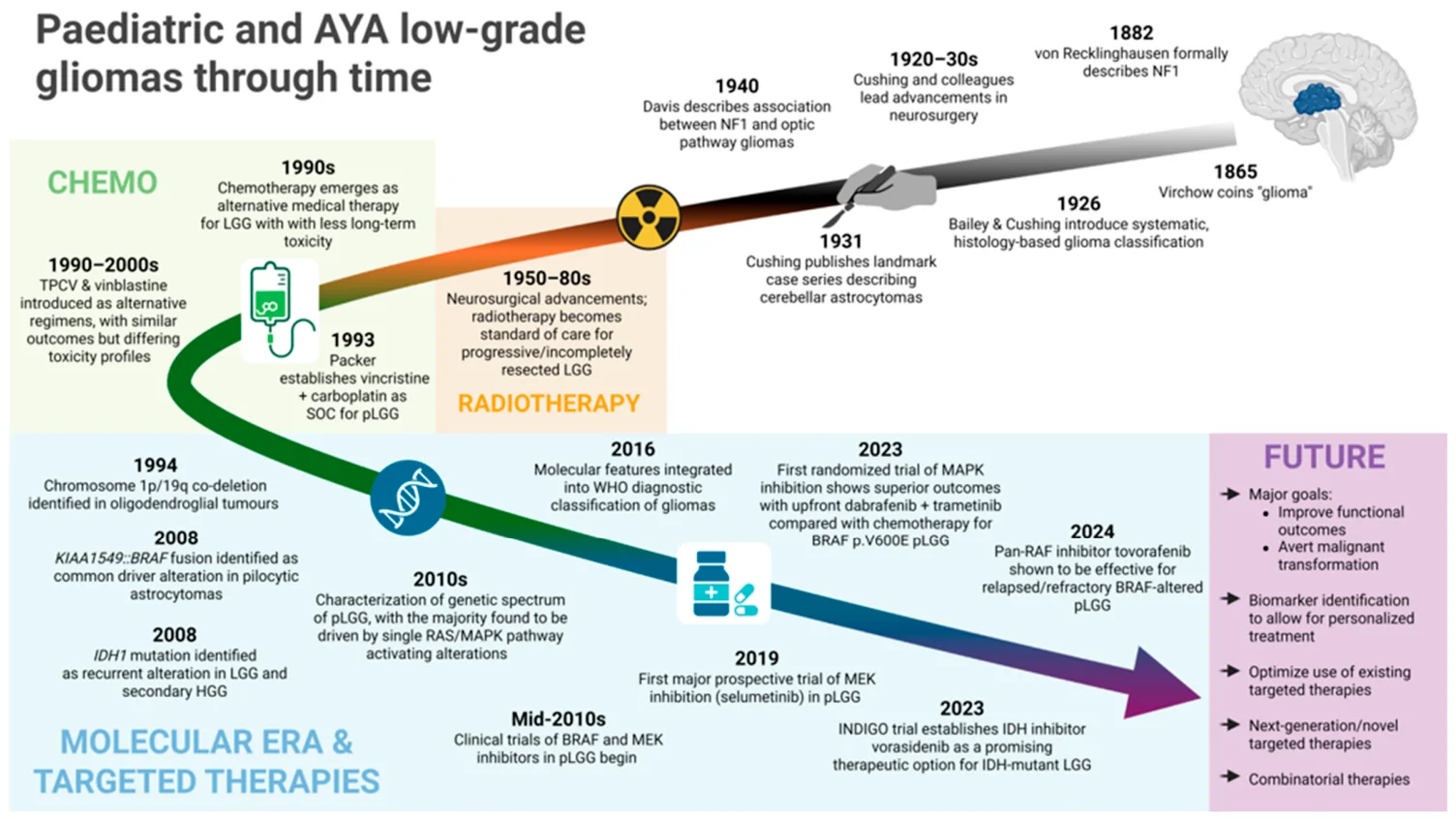
Timeline of major discoveries and advancements in treatment for paediatric and AYA low-grade gliomas. Abbreviations: LGG, low-grade glioma; pLGG, paediatric-type low-grade glioma; SOC, standard of care; TPCV, thioguanine, procarbazine, lomustine and vincristine. References: [3,31,36,39,42,44,103,104,105,106,107,108,109,110,111,112,113]. Created in BioRender. Burns, I. (2026) https://BioRender.com/b8dwlyh (accessed on 8 October 2026).

The molecular era has transformed our understanding of LGG and opened the door to novel therapies (Figure 2). Realizing the full potential of these advances will require continued progress across the spectrum from basic biology to clinical care to health care access, with the goal of improving the long-term functional outcomes of children, adolescents, and young adults with LGG.

## Figures and Tables

**Table 1:** Notable published phase II and III clinical trials for targeted therapies in clinical use for paediatric and AYA low-grade gliomas.

Type of Targeted Therapy	Drug and Trial Phase	Patient Population		Number of Patients	Radiologic Response	PFS	Reference
**BRAFi**	**Dabrafenib** Phase I/IIa	Recurrent, refractory, or progressive BRAF p.V600E pLGG (1–17 yo)		32	44% achieved >50% response (RANO)	1-year PFS 85%	[35]
**BRAFi + MEKi**	**Dabrafenib + trametinib** (versus chemotherapy) Phase II	BRAF p.V600E pLGG without previous medical therapy (1–17 yo)		73	47% achieved >50% response (RANO)	1-year PFS 67%	[36]
	**Dabrafenib + trametinib** Phase I/IIa	Relapsed/refractory BRAF p.V600E pLGG (1–17 yo)		36	25% achieved >50% response (RANO)	2-year PFS 80%	[37]
	**Dabrafenib + trametinib** Phase II	Recurrent or progressive BRAF p.V600E pLGG (>18 yo)		13	54% achieved >50% response (RANO)	Median PFS 9.2 months	[38]
**MEKi**	**Selumetinib** Phase II	Recurrent, refractory, or progressive pLGG (3–21 yo)	BRAF-aberrant PA	25	36% achieved >50% response	2-year PFS 70%	[39]
			BRAF-aberrant non-PA	23	22% achieved >50% response	2-year PFS 75%	[40]
			NF1-associated pLGG	25	40% achieved >50% response	2-year PFS 96%	[39]
			Optic pathway and hypothalamic pLGG (non-NF1)	25	24% achieved >50% response	2-year PFS 78%	[41]
			Non-NF1 non-BRAF-aberrant PA	14	7% achieved >50% response	2-year PFS 57%	[40]
			Unknown molecular status	26	27% achieved >50% response	2-year PFS 72%	[40]
	**Trametinib** Phase I/II	Relapsed/refractory BRAF p.V600E pLGG (1–17 yo)		13	15% achieved >50% response (RANO)	2-year PFS 50%	[37]
**Pan-RAFi**	**Tovorafenib** Phase II	Relapsed/refractory BRAF-altered pLGG (1–24 yo)		76	51% achieved >25% response (RAPNO)	Median PFS 13.8 months (RAPNO)	[42]
**IDHi**	**Vorasidenib** (versus placebo) Phase III	Residual/recurrent grade 2 IDH1/2-mutant diffuse gliomas (>12 yo; youngest patient in vorasidenib arm was 21 yo)		168	Tumour growth rate –1.3% (versus +14.4% with placebo)	2-year PFS 59%	[43,44]

Abbreviations: AYA, adolescent and young adult; BRAFi, BRAF inhibitor; MEKi, MEK inhibitor; Pan-RAFi, Pan-RAF inhibitor; IDHi, IDH inhibitor; yo, years old; PA, pilocytic astrocytoma; NF1, neurofibromatosis type 1; RANO, Response Assessment in Neuro-Oncology Criteria; RAPNO, Response Assessment in Paediatric Neuro-Oncology Criteria; PFS, progression-free survival. Number of patients refers to the number of patients treated with targeted therapy.

## Data Availability

No new data were created or analyzed in this study. Data sharing is not applicable to this article.

## References

[1] Ostrom Q.T., Gittleman H., de Blank P.M., Finlay J.L., Gurney J.G., McKean-Cowdin R., Stearns D.S., Wolff J.E., Liu M., Wolinsky Y. (2016). American Brain Tumor Association Adolescent and Young Adult Primary Brain and Central Nervous System Tumors Diagnosed in the United States in 2008–2012. Neuro-Oncol..

[2] Ostrom Q.T., Price M., Neff C., Cioffi G., Waite K.A., Kruchko C., Barnholtz-Sloan J.S. (2022). CBTRUS Statistical Report: Primary Brain and Other Central Nervous System Tumors Diagnosed in the United States in 2015–2019. Neuro-Oncol..

[3] Ryall S., Zapotocky M., Fukuoka K., Nobre L., Guerreiro Stucklin A., Bennett J., Siddaway R., Li C., Pajovic S., Arnoldo A. (2020). Integrated Molecular and Clinical Analysis of 1,000 Pediatric Low-Grade Gliomas. Cancer Cell.

[4] Yeo K.K., Alexandrescu S., Cotter J.A., Vogelzang J., Bhave V., Li M.M., Ji J., Benhamida J.K., Rosenblum M.K., Bale T.A. (2023). Multi-institutional study of the frequency, genomic landscape, and outcome of IDH-mutant glioma in pediatrics. Neuro-Oncol..

[5] Bennett J., Levine A.B., Nobre L., Negm L., Chung J., Fang K., Johnson M., Komosa M., Krumholtz S., Nunes N.M. (2025). A population-based analysis of the molecular landscape of glioma in adolescents and young adults reveals insights into gliomagenesis. Nat. Cancer.

[6] Armstrong G.T., Conklin H.M., Huang S., Srivastava D., Sanford R., Ellison D.W., Merchant T.E., Hudson M.M., Hoehn M.E., Robison L.L. (2011). Survival and long-term health and cognitive outcomes after low-grade glioma. Neuro-Oncol..

[7] Morin A., Allodji R., Kariyawasam D., Touraine P., Puget S., Beccaria K., De Carli E., Kieffer V., Rivollet S., Abbou S. (2024). Very long-term outcomes of pediatric patients treated for optic pathway gliomas: A longitudinal cohort study. Neuro-Oncol..

[8] Mistry M., Zhukova N., Merico D., Rakopoulos P., Krishnatry R., Shago M., Stavropoulos J., Alon N., Pole J.D., Ray P.N. (2015). BRAF mutation and CDKN2A deletion define a clinically distinct subgroup of childhood secondary high-grade glioma. J. Clin. Oncol..

[9] Bandopadhayay P., Bergthold G., London W.B., Goumnerova L.C., Morales La Madrid A., Marcus K.J., Guo D., Ullrich N.J., Robison N.J., Chi S.N. (2014). Long-term outcome of 4,040 children diagnosed with pediatric low-grade gliomas: An analysis of the Surveillance Epidemiology and End Results (SEER) database. Pediatr. Blood Cancer.

[10] Krishnatry R., Zhukova N., Guerreiro Stucklin A.S., Pole J.D., Mistry M., Fried I., Ramaswamy V., Bartels U., Huang A., Laperriere N. (2016). Clinical and treatment factors determining long-term outcomes for adult survivors of childhood low-grade glioma: A population-based study. Cancer.

[11] Wisoff J.H., Sanford R.A., Heier L.A., Sposto R., Burger P.C., Yates A.J., Holmes E.J., Kun L.E. (2011). Primary Neurosurgery for Pediatric Low-Grade Gliomas: A Prospective Multi-Institutional Study From the Children’s Oncology Group. Neurosurgery.

[12] Ris M.D., Leisenring W.M., Goodman P., Di C., Noll J., Levy W., Robison L.L., Armstrong G.T. (2019). Neuropsychological and socioeconomic outcomes in adult survivors of pediatric low-grade glioma. Cancer.

[13] Picariello S., Walters B., Bowman R., Jorgensen M., Slater O., O’Hare P., Spoudeas H.A., Aquilina K., Chang Y.-C., Cerbone M. (2025). Visual morbidity, long-term outcome and prognostic factors in infants and young children with optic pathway low-grade glioma. Neuro-Oncol. Pract..

[14] Ante Z., Babujee A., Liu N., McMahon J., Colpron M.-H., Koukoui B., McKeown T., Desjardins L., Wasserman J., Pechlivanoglou P. (2026). ID #276 Long-term medical and psychosocial outcomes of a population-based, molecularly characterized pediatric low-grade glioma survivor cohort. Neuro-Oncol. Pediatr..

[15] Rakotonjanahary J., Gravier N., Lambron J., De Carli E., Toulgoat F., Delion M., Pellier I., Rialland X. (2019). Long-term visual acuity in patients with optic pathway glioma treated during childhood with up-front BB-SFOP chemotherapy—Analysis of a French pediatric historical cohort. PLoS ONE.

[16] Wan M.J., Ullrich N.J., Manley P.E., Kieran M.W., Goumnerova L.C., Heidary G. (2016). Long-term visual outcomes of optic pathway gliomas in pediatric patients without neurofibromatosis type 1. J. Neuro-Oncol..

[17] Levine A.B., Bennett J., Patil P., Burns I., Siddaway R., Li C., Haizel-Cobbina J., Rana M., Yuditskiy R., Son A. (2026). Integrated clinical and molecular landscape of disseminated pediatric low-grade glioma. Neuro-Oncol..

[18] Burns I., Rutten C., Bennett J., Pai V., Pak A., Liu Y., Das A., Ramaswamy V., Bartels U., Huang A. (2026). ID #284 The impact of driver alterations on the long-term outcomes of paediatric low-grade gliomas treated with upfront chemotherapy. Neuro-Oncol. Pediatr..

[19] Lim-Fat M.J., Bennett J., Ostrom Q., Touat M., Franceschi E., Schulte J., Bindra R.S., Fangusaro J., Dhall G., Nicholson J. (2025). Central nervous system tumors in adolescents and young adults: A Society for Neuro-Oncology Consensus Review on diagnosis, management, and future directions. Neuro-Oncol..

[20] Hervey-Jumper S.L., Zhang Y., Phillips J.J., Morshed R.A., Young J.S., McCoy L., Lafontaine M., Luks T., Ammanuel S., Kakaizada S. (2023). Interactive Effects of Molecular, Therapeutic, and Patient Factors on Outcome of Diffuse Low-Grade Glioma. J. Clin. Oncol..

[21] Merchant T.E., Conklin H.M., Wu S., Lustig R.H., Xiong X. (2009). Late effects of conformal radiation therapy for pediatric patients with low-grade glioma: Prospective evaluation of cognitive, endocrine, and hearing deficits. J. Clin. Oncol..

[22] Grill J., Couanet D., Cappelli C., Habrand J.L., Rodriguez D., Sainte-Rose C., Kalifa C. (1999). Radiation-induced cerebral vasculopathy in children with neurofibromatosis and optic pathway glioma. Ann. Neurol..

[23] Packer R.J., Ater J., Allen J., Phillips P., Geyer R., Nicholson H.S., Jakacki R., Kurczynski E., Needle M., Finlay J. (1997). Carboplatin and vincristine chemotherapy for children with newly diagnosed progressive low-grade gliomas. J. Neurosurg..

[24] Laithier V., Grill J., Le Deley M.C., Ruchoux M.M., Couanet D., Doz F., Pichon F., Rubie H., Frappaz D., Vannier J.P. (2003). Progression-free survival in children with optic pathway tumors: Dependence on age and the quality of the response to chemotherapy—Results of the first French prospective study for the French Society of Pediatric Oncology. J. Clin. Oncol..

[25] Ater J.L., Zhou T., Holmes E., Mazewski C.M., Booth T.N., Freyer D.R., Lazarus K.H., Packer R.J., Prados M., Sposto R. (2012). Randomized study of two chemotherapy regimens for treatment of low-grade glioma in young children: A report from the Children’s Oncology Group. J. Clin. Oncol..

[26] Ater J.L., Xia C., Mazewski C.M., Booth T.N., Freyer D.R., Packer R.J., Sposto R., Vezina G., Pollack I.F. (2016). Nonrandomized comparison of neurofibromatosis type 1 and non-neurofibromatosis type 1 children who received carboplatin and vincristine for progressive low-grade glioma: A report from the Children’s Oncology Group. Cancer.

[27] Lassaletta A., Scheinemann K., Zelcer S.M., Hukin J., Wilson B.A., Jabado N., Carret A.S., Lafay-Cousin L., Larouche V., Hawkins C.E. (2016). Phase II Weekly Vinblastine for Chemotherapy-Naïve Children With Progressive Low-Grade Glioma: A Canadian Pediatric Brain Tumor Consortium Study. J. Clin. Oncol..

[28] Scheinemann K., Bartels U., Tsangaris E., Hawkins C., Huang A., Dirks P., Fried I., Bouffet E., Tabori U. (2011). Feasibility and efficacy of repeated chemotherapy for progressive pediatric low-grade gliomas. Pediatr. Blood Cancer.

[29] Burns I., Rutten C., Bennett J., Pai V., Liu A., Das A., Huang A., Ramaswamy V., Bartels U., McKeown T. (2026). ID #361 Superior tumour control with targeted therapy relative to conventional chemotherapy for BRAF-altered relapsed paediatric low-grade gliomas. Neuro-Oncol. Pediatr..

[30] Kandels D., Pietsch T., Bison B., Warmuth-Metz M., Thomale U.-W., Kortmann R.-D., Timmermann B., Hernáiz Driever P., Witt O., Schmidt R. (2020). Loss of efficacy of subsequent nonsurgical therapy after primary treatment failure in pediatric low-grade glioma patients—Report from the German SIOP-LGG 2004 cohort. Int. J. Cancer.

[31] Lassaletta A., Zápotocký M., Bouffet E. (2024). Chemotherapy in pediatric low-grade gliomas (PLGG). Child’s Nerv. Syst..

[32] Alshammari A.J.M., Hargrave D., Das A. (2026). RAS/MAPK inhibition in pediatric gliomas. Neuro-Oncol. Adv..

[33] Sait S.F., Bale T., Karajannis M.A. (2026). FGFR alterations and potential role of FGFR inhibitors in pediatric brain tumors. Neuro-Oncol. Adv..

[34] Mullins A., Bennett J., Yeo K.K., Lim-Fat M.J. (2026). Targeted therapies in adolescent and young adult patients with central nervous system tumors. Neuro-Oncol. Adv..

[35] Hargrave D.R., Bouffet E., Tabori U., Broniscer A., Cohen K.J., Hansford J.R., Geoerger B., Hingorani P., Dunkel I.J., Russo M.W. (2019). Efficacy and Safety of Dabrafenib in Pediatric Patients with BRAF V600 Mutation-Positive Relapsed or Refractory Low-Grade Glioma: Results from a Phase I/IIa Study. Clin. Cancer Res..

[36] Bouffet E., Hansford J.R., Garrè M.L., Hara J., Plant-Fox A., Aerts I., Locatelli F., van der Lugt J., Papusha L., Sahm F. (2023). Dabrafenib plus Trametinib in Pediatric Glioma with BRAF V600 Mutations. N. Engl. J. Med..

[37] Bouffet E., Geoerger B., Moertel C., Whitlock J.A., Aerts I., Hargrave D., Osterloh L., Tan E., Choi J., Russo M. (2023). Efficacy and Safety of Trametinib Monotherapy or in Combination With Dabrafenib in Pediatric BRAF V600-Mutant Low-Grade Glioma. J. Clin. Oncol..

[38] Subbiah V., Kreitman R.J., Wainberg Z.A., Gazzah A., Lassen U., Stein A., Wen P.Y., Dietrich S., de Jonge M.J.A., Blay J.Y. (2023). Dabrafenib plus trametinib in BRAFV600E-mutated rare cancers: The phase 2 ROAR trial. Nat. Med..

[39] Fangusaro J., Onar-Thomas A., Young Poussaint T., Wu S., Ligon A.H., Lindeman N., Banerjee A., Packer R.J., Kilburn L.B., Goldman S. (2019). Selumetinib in paediatric patients with BRAF-aberrant or neurofibromatosis type 1-associated recurrent, refractory, or progressive low-grade glioma: A multicentre, phase 2 trial. Lancet Oncol..

[40] Fangusaro J., Onar-Thomas A., Young Poussaint T., Lensing S., Ligon A.H., Lindeman N., Banerjee A., Kilburn L.B., Lenzen A., Pillay-Smiley N. (2025). A phase 2 PBTC study of selumetinib for recurrent/progressive pediatric low-grade glioma: Strata 2, 5, and 6 with long-term outcomes on strata 1, 3, and 4. Neuro-Oncol..

[41] Fangusaro J., Onar-Thomas A., Poussaint T.Y., Wu S., Ligon A.H., Lindeman N., Campagne O., Banerjee A., Gururangan S., Kilburn L.B. (2021). A phase II trial of selumetinib in children with recurrent optic pathway and hypothalamic low-grade glioma without NF1: A Pediatric Brain Tumor Consortium study. Neuro-Oncol..

[42] Kilburn L.B., Khuong-Quang D.-A., Hansford J.R., Landi D., van der Lugt J., Leary S.E.S., Driever P.H., Bailey S., Perreault S., McCowage G. (2024). The type II RAF inhibitor tovorafenib in relapsed/refractory pediatric low-grade glioma: The phase 2 FIREFLY-1 trial. Nat. Med..

[43] Cloughesy T.F., van den Bent M.J., Touat M., Blumenthal D.T., Peters K.B., Ellingson B.M., Clarke J.L., Mendez J., Yust-Katz S., Welsh L. (2025). Vorasidenib in IDH1-mutant or IDH2-mutant low-grade glioma (INDIGO): Secondary and exploratory endpoints from a randomised, double-blind, placebo-controlled, phase 3 trial. Lancet Oncol..

[44] Mellinghoff I.K., Bent M.J., Blumenthal D.T., Touat M., Peters K.B., Clarke J., Mendez J., Yust-Katz S., Welsh L., Mason W.P. (2023). Vorasidenib in IDH1- or IDH2-Mutant Low-Grade Glioma. N. Engl. J. Med..

[45] Nicolaides T., Nazemi K.J., Crawford J., Kilburn L., Minturn J., Gajjar A., Gauvain K., Leary S., Dhall G., Aboian M. (2020). Phase I study of vemurafenib in children with recurrent or progressive BRAFV600E mutant brain tumors: Pacific Pediatric Neuro-Oncology Consortium study (PNOC-002). Oncotarget.

[46] Nobre L., Zapotocky M., Ramaswamy V., Ryall S., Bennett J., Alderete D., Balaguer Guill J., Baroni L., Bartels U., Bavle A. (2020). Outcomes of BRAF V600E Pediatric Gliomas Treated With Targeted BRAF Inhibition. JCO Precis. Oncol..

[47] Suh J.K., Koh K.-N. (2026). Current Perspectives on Pediatric Low-Grade Gliomas: Focus on BRAF and MEK Inhibition. Brain Tumor Res. Treat..

[48] Karajannis M.A., Legault G., Fisher M.J., Milla S.S., Cohen K.J., Wisoff J.H., Harter D.H., Goldberg J.D., Hochman T., Merkelson A. (2014). Phase II study of sorafenib in children with recurrent or progressive low-grade astrocytomas. Neuro-Oncol..

[49] Barbato M.I., Nashed J., Bradford D., Ren Y., Khasar S., Miller C.P., Zolnik B.S., Zhao H., Li Y., Bi Y. (2024). FDA Approval Summary: Dabrafenib in Combination with Trametinib for BRAFV600E Mutation-Positive Low-Grade Glioma. Clin. Cancer Res..

[50] Wen P.Y., Stein A., van den Bent M., De Greve J., Wick A., de Vos F., von Bubnoff N., van Linde M.E., Lai A., Prager G.W. (2022). Dabrafenib plus trametinib in patients with BRAF(V600E)-mutant low-grade and high-grade glioma (ROAR): A multicentre, open-label, single-arm, phase 2, basket trial. Lancet Oncol..

[51] Erker C., Vanan M.I., Larouche V., Nobre L., Cacciotti C., Vairy S., Zelcer S., Fleming A., Bouffet E., Jabado N. (2024). Canadian Consensus for Treatment of BRAF V600E Mutated Pediatric and AYA Gliomas. Curr. Oncol..

[52] Gascon B., Gauvreau C., Yang A., Bennett J., Nobre L., Tabori U., Hawkins C., Tsang D., Pechlivanoglou P., Denburg A. (2026). Cost-Effectiveness of Dabrafenib Plus Trametinib Versus Standard Chemotherapy in BRAFV600E-Mutant Pediatric Low-Grade Glioma. JCO Oncol. Pract..

[53] Fangusaro J., Onar-Thomas A., Wu S., Lensing S., Packer R., Kilburn L., Qaddoumi I., Dhall G., Pollack I., Lenzen A. (2026). ID #98 A Phase 2 Re-treatment Study of Selumetinib for Recurrent/Progressive Pediatric Low-Grade Glioma (pLGG): A Pediatric Brain Tumor Consortium (PBTC) Study, PBTC-029C. Neuro-Oncol. Pediatr..

[54] Green K., Vardon A., Sidra J., Mankad K., Bailey S., Ritzmann T., Thust S., Wilson S., Ganosi V., Gagen R. (2026). A nationwide evaluation of mapk inhibitors trametinib and dabrafenib for paediatric low-grade glioma in the United Kingdom: Safety, efficacy, visual morbidity, and outcomes. Neuro-Oncol. Pediatr..

[55] Manoharan N., Choi J., Chordas C., Zimmerman M.A., Scully J., Clymer J., Filbin M., Ullrich N.J., Bandopadhayay P., Chi S.N. (2020). Trametinib for the treatment of recurrent/progressive pediatric low-grade glioma. J. Neuro-Oncol..

[56] Tsai J.W., Choi J.J., Ouaalam H., Murillo E.A., Yeo K.K., Vogelzang J., Sousa C., Woods J.K., Ligon K.L., Warfield S.K. (2023). Integrated response analysis of pediatric low-grade gliomas during and after targeted therapy treatment. Neuro-Oncol. Adv..

[57] Selt F., Van Tilburg C.M., Bison B., Sievers P., Harting I., Ecker J., Pajtler K.W., Sahm F., Bahr A., Simon M. (2020). Response to trametinib treatment in progressive pediatric low-grade glioma patients. J. Neuro-Oncol..

[58] Perreault S., Larouche V., Eltaani N., Tabori U., Hawkins C., Lippé S., Ellezam B., Décarie J.-C., Kiaei D., Ospina L. (2026). ID #515 TRAM-01: A Phase 2 study of trametinib for patients with pediatric low grade glioma with activation of the MAPK/ERK pathway. Neuro-Oncol. Pediatr..

[59] Robison N., Pauly J., Malvar J., Gardner S., Allen J., Margol A., MacDonald T., Bendel A., Kilburn L., Cluster A. (2022). LTBK-04. LATE BREAKING ABSTRACT: MEK162 (binimetinib) in children with progressive or recurrent low-grade glioma: A multi-institutional phase II and target validation study. Neuro-Oncol..

[60] Robinson G.W., Vinitsky A., Bag A.K., Chiang J., Li Q., Lin T., Hoehn M.E., Edwards A., Storment D., Singh A. (2024). Lgg-53. Results from the Phase 1 and Phase 1 Expansion Cohorts of Sj901: A Phase 1/2 Trial of Single-Agent Mirdametinib (Pd-0325901) in Children, Adolescents, and Young Adults with Low-Grade Glioma. Neuro-Oncol..

[61] Apfelbaum A.A., Morin E., Sturm D., Ayoub G., DiGiacomo J., Bahadur S., Chandarana B., Power P.C., Cusick M.M., Novikov D. (2025). A diverse landscape of FGFR alterations and co-mutations suggests potential therapeutic strategies in pediatric low-grade gliomas. Nat. Commun..

[62] Quang D., Landi D., Kline C., Hargrave D., Leary S., van der Lugt J., Mueller S., Nysom K., Perreault S., Waanders A. (2026). ID #832 FIREFLY-1 3-Year Results: Impact of Prior Therapy on Tovorafenib Activity in BRAF-Altered Pediatric Low-Grade Glioma. Neuro-Oncol. Pediatr..

[63] Singh S., Bradford D., Chatterjee S., Li X., Aungst S.L., Skinner A.M., Miller C.P., Kim-McOlash S., Fourie Zirkelbach J., Xiong Y. (2025). FDA Approval Summary: Tovorafenib for Relapsed or Refractory BRAF-Altered Pediatric Low-Grade Glioma. Clin. Cancer Res..

[64] Sigaud R., Rösch L., Gatzweiler C., Benzel J., von Soosten L., Peterziel H., Selt F., Najafi S., Ayhan S., Gerloff X.F. (2023). The first-in-class ERK inhibitor ulixertinib shows promising activity in mitogen-activated protein kinase (MAPK)-driven pediatric low-grade glioma models. Neuro-Oncol..

[65] Vo K.T., Sabnis A.J., Williams P.M., Roy-Chowdhuri S., Patton D.R., Coffey B., Reid J.M., Piao J., Saguilig L., Alonzo T.A. (2024). Phase II Study of Ulixertinib in Children and Young Adults With Tumors Harboring Activating Mitogen-Activated Protein Kinase Pathway Alterations: APEC1621J of the National Cancer Institute-Children’s Oncology Group Pediatric MATCH Trial. JCO Precis. Oncol..

[66] Hargrave D., Bowers D.C., Goldman S., Liu G.T., Huang J.T., van Santen H.M., Robison N.J., Zapotocky M., Bouffet E. (2026). Management of adverse events in patients with pediatric low-grade glioma treated with MAPK-directed therapies: Delphi consensus recommendations and unmet needs. Neuro-Oncol. Pract..

[67] Peters K.B. (2026). New and Emerging Therapies for Patients with Low-Grade Glioma. CNS Drugs.

[68] Kline C., Wright K.D., Chi S.N., Landi D.B., van der Lugt J., Leary S.E.S., Abdelbaki M.S., Bailey S., Nysom K., Khuong-Quang D.-A. (2026). Growth recovery in patients with BRAF-altered pediatric low-grade glioma following treatment with tovorafenib. Neuro-Oncol. Pediatr..

[69] Haas-Kogan D.A., Aboian M.S., Minturn J.E., Leary S.E.S., Abdelbaki M.S., Goldman S., Elster J.D., Kraya A., Lueder M.R., Ramakrishnan D. (2024). Everolimus for Children With Recurrent or Progressive Low-Grade Glioma: Results From the Phase II PNOC001 Trial. J. Clin. Oncol..

[70] Wright K.D., Yao X., London W.B., Kao P.C., Gore L., Hunger S., Geyer R., Cohen K.J., Allen J.C., Katzenstein H.M. (2021). A POETIC Phase II study of continuous oral everolimus in recurrent, radiographically progressive pediatric low-grade glioma. Pediatr. Blood Cancer.

[71] Ullrich N.J., Prabhu S.P., Reddy A.T., Fisher M.J., Packer R., Goldman S., Robison N.J., Gutmann D.H., Viskochil D.H., Allen J.C. (2020). A phase II study of continuous oral mTOR inhibitor everolimus for recurrent, radiographic-progressive neurofibromatosis type 1-associated pediatric low-grade glioma: A Neurofibromatosis Clinical Trials Consortium study. Neuro-Oncol..

[72] de la Fuente M.I., Colman H., Rosenthal M., Van Tine B.A., Levacic D., Walbert T., Gan H.K., Vieito M., Milhem M.M., Lipford K. (2023). Olutasidenib (FT-2102) in patients with relapsed or refractory IDH1-mutant glioma: A multicenter, open-label, phase Ib/II trial. Neuro-Oncol..

[73] Lin M.D., Tsai A.C.Y., Abdullah K.G., McBrayer S.K., Shi D.D. (2024). Treatment of IDH-mutant glioma in the INDIGO era. npj Precis. Oncol..

[74] Nysom K., Kilburn L.B., Leary S.E.S., Landi D.B., de Vos-Kerkhof E., Perreault S., Witt O., Ziegler D.S., Hernáiz Driever P., Franson A.T. (2025). Radiographic and visual response to the type II RAF inhibitor tovorafenib in children with relapsed/refractory optic path-way glioma in the FIREFLY-1 trial. Neuro-Oncol..

[75] Fangusaro J., Witt O., Hernáiz Driever P., Bag A.K., de Blank P., Kadom N., Kilburn L., Lober R.M., Robison N.J., Fisher M.J. (2020). Response assessment in paediatric low-grade glioma: Recommendations from the Response Assessment in Pediatric Neuro-Oncology (RAPNO) working group. Lancet Oncol..

[76] Fisher M.J., Avery R.A., Allen J.C., Ardern-Holmes S.L., Bilaniuk L.T., Ferner R.E., Gutmann D.H., Listernick R., Martin S., Ullrich N.J. (2013). Functional outcome measures for NF1-associated optic pathway glioma clinical trials. Neurology.

[77] Lim-Fat M.J., Cotter J.A., Touat M., Vogelzang J., Sousa C., Pisano W., Geduldig J., Bhave V., Driver J., Kao P.C. (2024). A comparative analysis of IDH-mutant glioma in pediatric, young adult, and older adult patients. Neuro-Oncol..

[78] de Lande R.S.V., Maurice-Stam H., Marchal J.P., Vuurden D.G.V., Vandertop W.P., Grootenhuis M.A., Schouten-van Meeteren A.Y.N. (2019). Adaptive behavior impaired in children with low-grade glioma. Pediatr. Blood Cancer.

[79] Gnekow A.K., Walker D.A., Kandels D., Picton S., Perilongo G., Grill J., Stokland T., Sandstrom P.E., Warmuth-Metz M., Pietsch T. (2017). A European randomised controlled trial of the addition of etoposide to standard vincristine and carboplatin induction as part of an 18-month treatment programme for childhood (≤16 years) low grade glioma—A final report. Eur. J. Cancer.

[80] Levine A.B., Nobre L., Das A., Milos S., Bianchi V., Johnson M., Fernandez N.R., Stengs L., Ryall S., Ku M. (2024). Immuno-oncologic profiling of pediatric brain tumors reveals major clinical significance of the tumor immune microenvironment. Nat. Commun..

[81] Andrade A.F., Sigaud R., Puligandla E., Liu B., Karimi E., Annett A., Rezanejad M., Jawhar W., Taylor R., Cao Y. (2025). A spatial map of MAPK-activated immunosuppressive myeloid populations in pediatric low-grade glioma. Nat. Immunol..

[82] Zahedi S., Riemondy K., Liu T., Griesinger A.M., Donson A.M., Apfelbaum A.A., Fu R., Grandvallet Contreras J., Crespo M., DeSisto J. (2025). Multi-pronged analysis of pediatric low-grade glioma and ganglioglioma reveals a unique tumor microenvironment associated with BRAF alterations. Brain Pathol..

[83] Sigaud R., Albert T.K., Hess C., Hielscher T., Winkler N., Kocher D., Walter C., Münter D., Selt F., Usta D. (2023). MAPK inhibitor sensitivity scores predict sensitivity driven by the immune infiltration in pediatric low-grade gliomas. Nat. Commun..

[84] Kocher D., Cao L., Guiho R., Langhammer M., Lai Y.L., Becker P., Hamdi H., Friedel D., Selt F., Vonhören D. (2024). Rebound growth of BRAF mutant pediatric glioma cells after MAPKi withdrawal is associated with MAPK reactivation and secretion of microglia-recruiting cytokines. J. Neuro-Oncol..

[85] Haist M., Stege H., Kuske M., Bauer J., Klumpp A., Grabbe S., Bros M. (2023). Combination of immune-checkpoint inhibitors and targeted therapies for melanoma therapy: The more, the better?. Cancer Metastasis Rev..

[86] Gorodezki D., Schuhmann M.U., Ebinger M., Schittenhelm J. (2024). Dissecting the Natural Patterns of Progression and Senescence in Pediatric Low-Grade Glioma: From Cellular Mechanisms to Clinical Implications. Cells.

[87] Selt F., Sigaud R., Valinciute G., Sievers P., Zaman J., Alcon C., Schmid S., Peterziel H., Tsai J.W., Guiho R. (2023). BH3 mimetics targeting BCL-XL impact the senescent compartment of pilocytic astrocytoma. Neuro-Oncol..

[88] Plant-Fox A.S., Tabori U. (2024). Future perspective of targeted treatments in pediatric low-grade glioma (pLGG): The evolution of standard-of-care and challenges of a new era. Child’s Nerv. Syst..

[89] Kilburn L.B., Hoffman L.M., Levy J.M., Billups C., Vinitsky A., McCourt E., Poussaint T.Y., Vibhakar R., Sait S.F., Baxter P. Dabrafenib, trametinib, and hydroxychloroquine (HCQ) and trametinib and HCQ demonstrate safety and efficacy in pediatric and young adult patients with BRAFV600E and BRAF fusion/duplication positive recurrent or progressive low grade gliomas: Results from a Pediatric Brain Tumor Consortium clinical trial (PBTC-055). Proceedings of the ISPNO 2026.

[90] Albert N.L., Galldiks N., Ellingson B.M., van den Bent M.J., Chang S.M., Cicone F., de Groot J., Koh E.S., Law I., Le Rhun E. (2024). PET-based response assessment criteria for diffuse gliomas (PET RANO 1.0): A report of the RANO group. Lancet Oncol..

[91] Albert N.L., Furtner J., van den Bent M.J., Preusser M. (2024). The potential of amino acid PET imaging for prediction and monitoring of vorasidenib response in IDH-mutant gliomas. Neuro-Oncol..

[92] Nobre L., Nakano Y., Burns I., Siddaway R., Zápotocky M., Johnson M., Rana M., Li C., Lyn R.K., Yuditskiy R. (2026). Tumor-derived cell-free DNA detected in cerebrospinal fluid enables minimally invasive profiling of pediatric brain tumors. J. Clin. Investig..

[93] Sole V.M., Elia G., Alessandro P., Marco P., Gianluca P., Pietro F., Gianluigi Z., Gabriele G., Andrea B. (2026). Cerebral gliomas in adolescents and young adults: A scoping review of evidence gaps in diagnosis, treatment, and prognosis. Eur. J. Pediatr..

[94] Murciano-Goroff Y.R., Uppal M., Chen M., Harada G., Schram A.M. (2024). Basket Trials: Past, Present, and Future. Annu. Rev. Cancer Biol..

[95] Rizzo D., Ruggiero A., Amato M., Maurizi P., Riccardi R. (2016). BRAF and MEK inhibitors in pediatric glioma: New therapeutic strategies, new toxicities. Expert Opin. Drug Metab. Toxicol..

[96] O’Hare P., Cooney T., de Blank P., Gutmann D.H., Kieran M., Milde T., Fangusaro J., Fisher M., Avula S., Packer R. (2024). Resistance, rebound, and recurrence regrowth patterns in pediatric low-grade glioma treated by MAPK inhibition: A modified Delphi approach to build international consensus-based definitions-International Pediatric Low-Grade Glioma Coalition. Neuro-Oncol..

[97] Dave B., Machado M., Siddaway R., Massey N., Suthakaran A., Chan A., Li C., Tsang D.S., Das S., Dirks P. (2025). Virtual CNS Tumor Board Rounds Within the Canadian Adolescent and Young Adult Neuro-Oncology Network. JCO Precis. Oncol..

[98] Barbato M.I., Barone A.K., Aungst S.L., Miller C.P., Ananthula S., Bi Y., Yang Y., Li X., Xiong Y., Fan J. (2025). FDA Approval Summary: Vorasidenib for IDH-Mutant Grade 2 Astrocytoma or Oligodendroglioma Following Surgery. Clin. Cancer Res..

[99] Kinori M., Armarnik S., Listernick R., Charrow J., Zeid J.L. (2021). Neurofibromatosis Type 1-Associated Optic Pathway Glioma in Children: A Follow-Up of 10 Years or More. Am. J. Ophthalmol..

[100] Sengupta D., Ringel A., Pak A., Liu Y., Bennett J., Das A., Huang A., Ramaswamy V., Bartels U., McKeown T. (2026). ID #394 Superior tumor control using weaning MEK inhibitors for RAS/MAPK altered pediatric low-grade glioma (pLGG). Neuro-Oncol. Pediatr..

[101] Borgenvik A., Misek S.A., Christen D., Kyrila G., Zhang A., Reel S., Boisvert M., Cai K.Y., Kovarzin A., Li H. (2026). O-mannosylation and protein maturation checkpoints represent therapeutic opportunities in BRAF fusion protein oncogenesis. Cancer Discov..

[102] Rossi J., Picca A., Santonocito O.S., Schembari S., Testaverde L., Sanson M., Berzero G., Di Stefano A.L. (2025). Advances in the management of patients with IDH-mutant glioma. Oncologist.

[103] Ishaque A.H., Das S. (2024). Cutting Through History: The Evolution of Glioblastoma Surgery. Curr. Oncol..

[104] Antônio J.R., Goloni-Bertollo E.M., Trídico L.A. (2013). Neurofibromatosis: Chronological history and current issues. An. Bras. Dermatol..

[105] Parsons D.W., Jones S., Zhang X., Lin J.C., Leary R.J., Angenendt P., Mankoo P., Carter H., Siu I.M., Gallia G.L. (2008). An integrated genomic analysis of human glioblastoma multiforme. Science.

[106] Packer R.J., Lange B., Ater J., Nicholson H.S., Allen J., Walker R., Prados M., Jakacki R., Reaman G., Needles M.N. (1993). Carboplatin and vincristine for recurrent and newly diagnosed low-grade gliomas of childhood. J. Clin. Oncol..

[107] Jones D.T., Kocialkowski S., Liu L., Pearson D.M., Bäcklund L.M., Ichimura K., Collins V.P. (2008). Tandem duplication producing a novel oncogenic BRAF fusion gene defines the majority of pilocytic astrocytomas. Cancer Res..

[108] Cushing H. (1931). Experiences with the cerebellar astrocytoma. A clinical review of seventy-six cases. Surg. Gynecol. Obstet..

[109] Louis D.N., Perry A., Reifenberger G., von Deimling A., Figarella-Branger D., Cavenee W.K., Ohgaki H., Wiestler O.D., Kleihues P., Ellison D.W. (2016). The 2016 World Health Organization Classification of Tumors of the Central Nervous System: A summary. Acta Neuropathol..

[110] Reifenberger J., Reifenberger G., Liu L., James C.D., Wechsler W., Collins V.P. (1994). Molecular genetic analysis of oligodendroglial tumors shows preferential allelic deletions on 19q and 1p. Am. J. Pathol..

[111] Pfister S., Janzarik W.G., Remke M., Ernst A., Werft W., Becker N., Toedt G., Wittmann A., Kratz C., Olbrich H. (2008). BRAF gene duplication constitutes a mechanism of MAPK pathway activation in low-grade astrocytomas. J. Clin. Investig..

[112] McCutcheon I.E., Preul M.C. (2021). Historical Perspective on Surgery and Survival with Glioblastoma: How Far Have We Come?. World Neurosurg..

[113] Bailey P., Cushing H. (1926). A Classification of the Tumors of the Glioma Group on a Histogenetic Basis with a Correlated Study of Prognosis.

